# Author Correction: Reverse engineering synthetic antiviral amyloids

**DOI:** 10.1038/s41467-023-39293-9

**Published:** 2023-06-13

**Authors:** Emiel Michiels, Kenny Roose, Rodrigo Gallardo, Ladan Khodaparast, Laleh Khodaparast, Rob van der Kant, Maxime Siemons, Bert Houben, Meine Ramakers, Hannah Wilkinson, Patricia Guerreiro, Nikolaos Louros, Suzanne J. F. Kaptein, Lorena Itatí Ibañez, Anouk Smet, Pieter Baatsen, Shu Liu, Ina Vorberg, Guy Bormans, Johan Neyts, Xavier Saelens, Frederic Rousseau, Joost Schymkowitz

**Affiliations:** 1 VIB Center for Brain and Disease Research, Leuven, Belgium; 2 Switch Laboratory, Department of Cellular and Molecular Medicine, KU Leuven, Leuven, Belgium; 3 VIB Center for Medical Biotechnology, Ghent, Belgium; 4 Department of Biomedical Molecular Biology, Ghent University, Ghent, Belgium; 5 Laboratory for Radiopharmacy, Department of Pharmaceutical and Pharmacological Sciences, KU Leuven, Leuven, Belgium; 6 Laboratory of Virology and Chemotherapy, Rega Institute for Medical Research, Department of Microbiology, Immunology and Transplantation, KU Leuven, Leuven, Belgium; 7 Electron Microscopy Platform of VIB Bio Imaging Core, KU Leuven, Leuven, Belgium; 8 German Center for Neurodegenerative Diseases (DZNE e.V.), Bonn, Germany; 9 Rheinische Friedrich-Wilhelms-Universität Bonn, Bonn, Germany

**Keywords:** Proteins, Proteomics, Structural biology, Molecular engineering, Recombinant peptide therapy

Correction to: *Nature Communications* 10.1038/s41467-020-16721-8, published online 05 June 2020

In this article the grant number G045920N relating to Funds for Scientific Research Flanders was omitted. The original article has been corrected.

