# Supplementary material for: Reverse engineering synthetic antiviral amyloids
Source: Nat Commun. 2020 Jun 5;11:2832. doi: 10.1038/s41467-020-16721-8 (PMC7275043; doi:10.1038/s41467-020-16721-8)
Supplement: Supplementary file 1 — Supplementary Information [file 41467_2020_16721_MOESM1_ESM.pdf]

Reverse engineering synthetic antiviral amyloids

Michiels et al.

Supplementary Figures

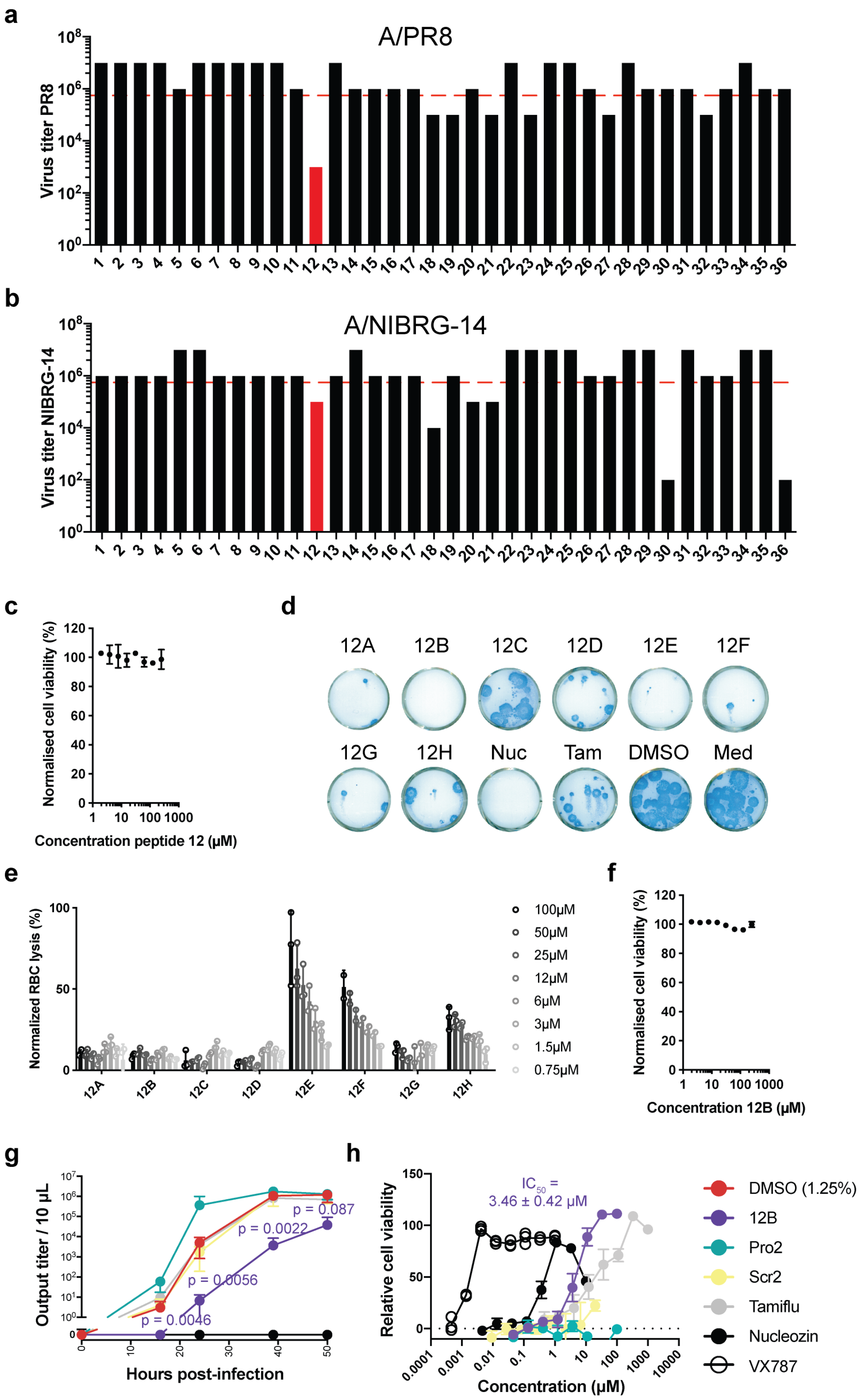

Supplementary Figure 1 – Identification and optimization of an amyloid-based anti-influenza A peptide. **(a-b)** Amount of newly produced virus of influenza A/PR8 (a) or A/NIBRG-14 (b) after peptide treatment as determined by TCID50 titration. Dashed line represents average viral titers of buffer-treated cells and peptide 12 is highlighted in red. **(c)** Dose-dependent toxicity of peptide 12, after 24-hours incubation on MDCK cells. Data is normalized to buffer-treated (100% viability) and 0.1% triton-treated cells (0% viability) and the mean ± SD is shown (n = 3 independent experiments). **(d)** Plaque-size reduction assay of MDCK cells treated with 10 µM peptide (Supplementary Table 2), Tamiflu (100 µM), Nucleozin (10 µM), DMSO (1%) or cell medium, 2 hours prior to virus infection with influenza A/PR8. **(e)** Red Blood Cell (RBC) lysis one hour after peptide addition (grey scale represents final peptide concentrations). Data is normalized to buffer-treated cells (0% lysis) and 0.1% triton-treated cells (100% lysis) and the mean values ± SD are shown (n = 3 independent experiments). **(f)** Dose-dependent toxicity of peptide 12B, after 24-hours incubation on HEK 293T cells. Data is normalized to buffer-treated (100% viability) and 0.1% triton-treated cells (0% viability) and the mean ± SD is shown (n = 3 independent experiments). **(g)** Multicycle replication of influenza A/PR8 in MDCK cells. Cells were infected, supernatants were collected and checked for amount of virus at different time points post infection. Data represent mean values of virus titer per 10 µL supernatant ± SD (n = 4 independent experiments, statistics: one-way ANOVA with multiple comparison to DMSO control). **(h)** Cytopathic effects of influenza A/PR8 replication on MCDK cells. Cells were infected and treated with 1.25% DMSO, 10 µM peptide, 100 µM Tamiflu, 10 µM Nucleozin or 1 µM VX-787. After 72 hours, cytopathic effects were quantified by assaying cell viability. Data are normalized to non-infected (100% viability) and DMSO-treated, infected cells (0% viability) and shown as mean values ± SD (n = 4 independent experiments).

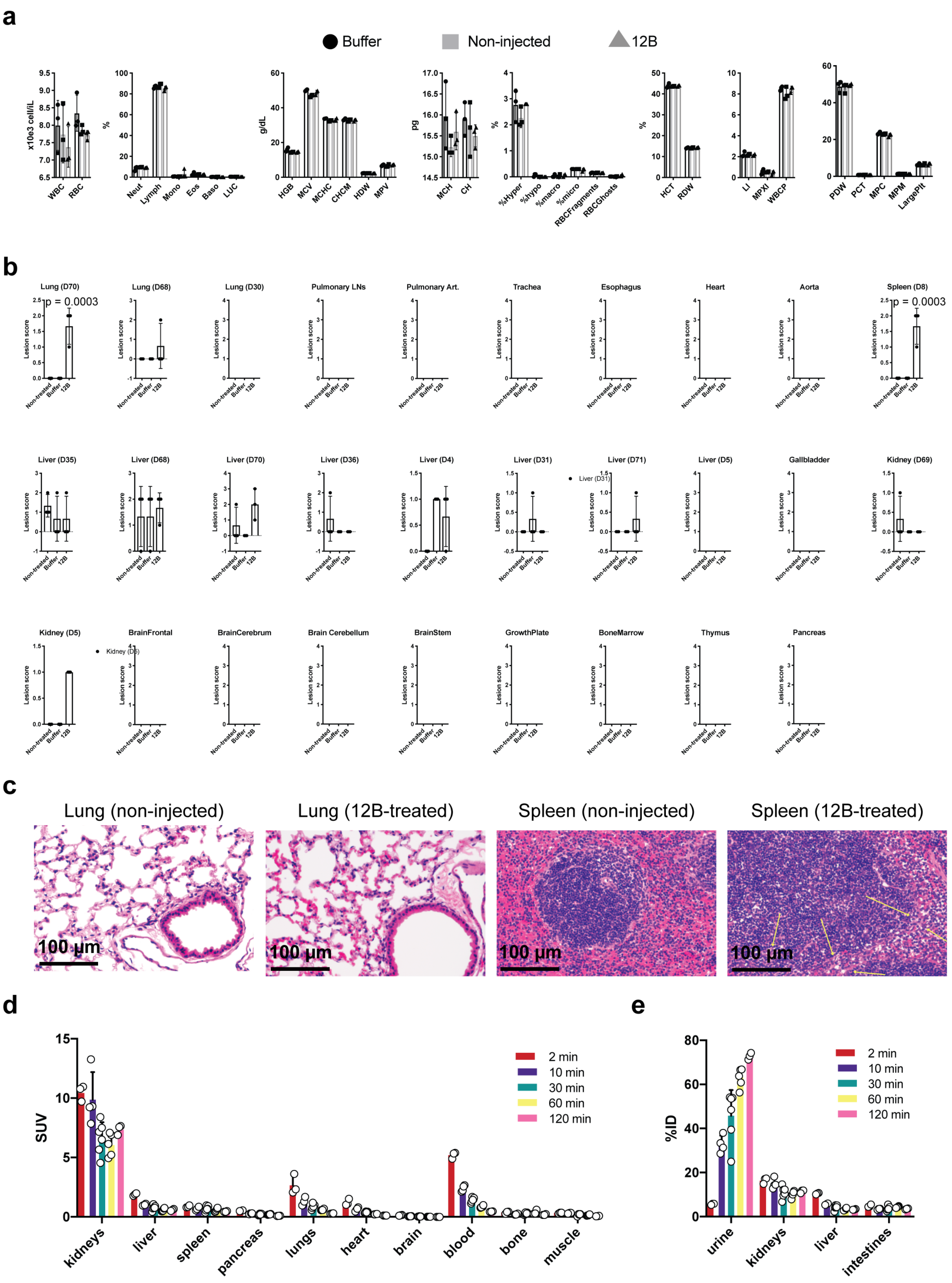

Supplementary Figure 2 – Peptide 12B in vivo toxicity and biodistribution. **(a)** Hematological analysis of the blood isolated from mice, treated daily for two weeks with 10 mg/kg peptide 12B or buffer. Mean values ± SD is shown (n = 12 mice over 3 independent experiments, statistics: one-way ANOVA). **(b)** Lesion scores of all organs studied in this toxicity study (details in **Supplementary Note 1**). Data represent mean lesions score ± SD (n = 12 mice over 3 independent experiments, statistics: one-way ANOVA with multiple comparison to non-

treated condition). **(c)** Hematoxylin and Eosin (H&E) staining of the two organs (lung and spleen) that showed minor lesions upon peptide treatment. Yellow arrows indicate splenic lymphoreticular cell infiltration. **(d)** *Ex vivo* biodistribution study of [^{68}Ga]Ga-NODAGA-PEG_2-12B in an influenza A/PR8 infection mouse model. The figure shows the relative peptide concentrations per organ (SUV, standardized uptake value), at different time points after peptide injection (color scales). Data are expressed as mean ± SD (n = 6 independent experiments for time points 2, 10 and 30 minutes and n = 3 independent experiments for 60 and 120 minutes). **(e)** The general peptide distribution (%ID, percentage injected dose) at different time points (color scales) to provide information about the clearance of the peptide. Data are expressed as mean ± SD (n = 6 independent experiments for time points 2, 10 and 30 minutes and n = 3 independent experiments for 60 and 120 minutes). Abbreviations: WBC, White Blood Cells. RBC, Red Blood Cells. Neut, Neutrophil percent. Lymph, Lymphocyte percent. Mono, Monocyte percent. Eos, Eosinophil percent. Baso, Basophil percent. LUC, Large unstained cells. HGB, Hemoglobin concentration. MCV, Mean Corpuscular Volume. MCHC, Mean Corpuscular Hemoglobin Concentration. CHCM, cell hemoglobin concentration mean. HDW, hemoglobin distribution width. MPV, Mean Platelet Volume. MCH, Mean Corpuscular Hemoglobin. CH, cellular hemoglobin content (mean of RBC cellular hemoglobin histogram). Hyper, hyperchromia. Hypo, hypochromia. Macro, macrocytosis. Micro, microcytosis. HCT, Hematocrit. RDW, red cell distribution width. LI, lobularity index. MPXI, myeloperoxidase index. WBCP, white blood cell count by peroxidase. PDW, Platelet Distribution Width. PCT, Plateletcrit. MPC, mean platelet component. MPM, malignant pleural mesothelioma. LargePlt, large platelets.

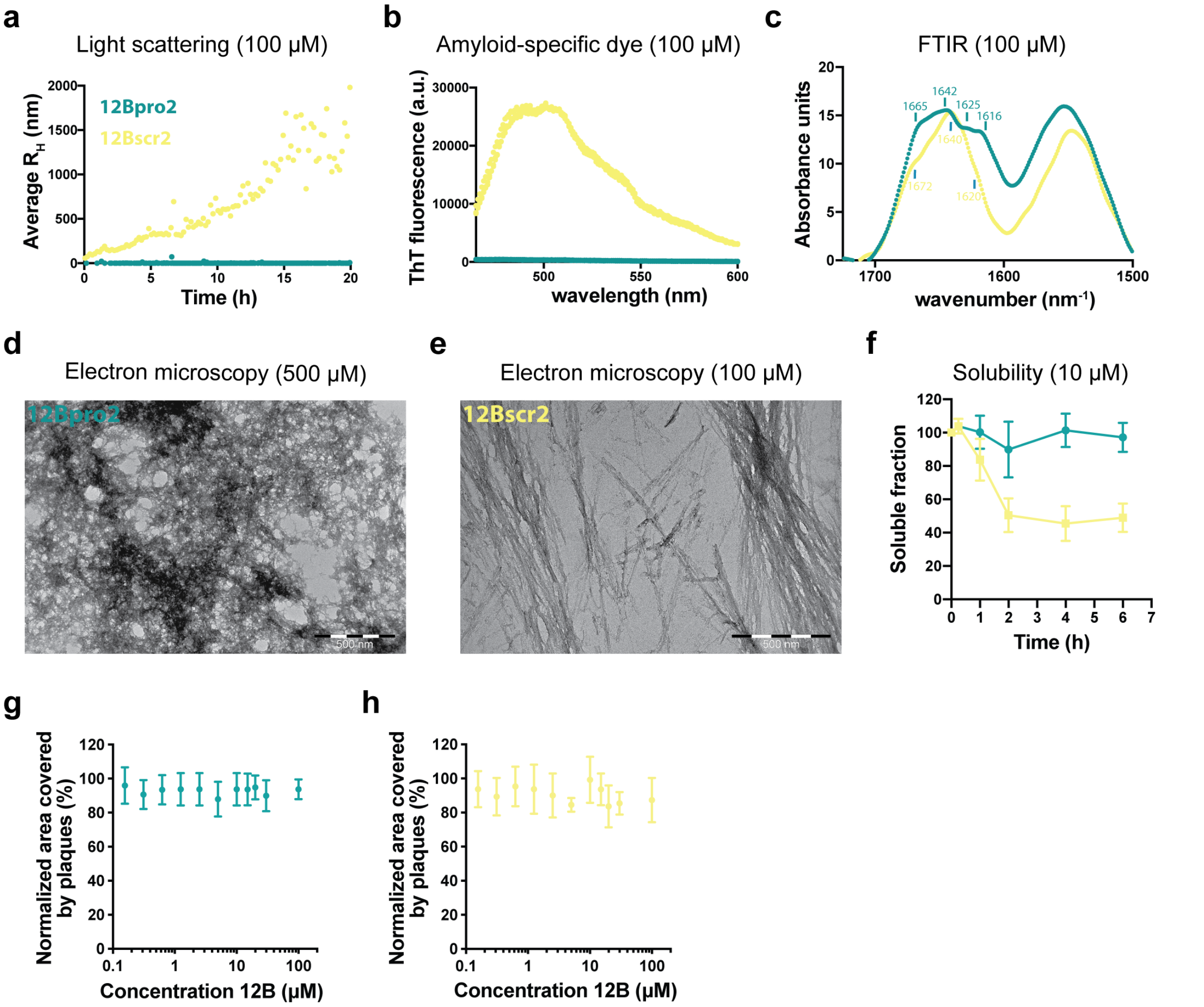

Supplementary Figure 3 – Amyloidogenic behavior of control peptides 12Bpro2 and 12Bscr2. **(a)** Hydrodynamic radius (R_H) calculated from the regularization fit of DLS data of peptides 12Bpro2 and 12Bscr2 (100 µM) over time. **(b)** Thioflavin-T emission spectrum after excitation at 440 nm of peptides 12Bpro2 and 12Bscr2 (100 µM) 5 minutes after solubilization (n = 3). **(c)** FTIR spectra of 12Bpro2 and 12Bscr2 (100 µM) 5 minutes after solubilization. **(d-e)** TEM of 500 µM peptide 12Bpro2 incubated for 1 week at room temperature (100 µM of peptide 12Bpro2 did not aggregate) and 100 µM peptide 12Bscr2 incubated for 24 hours, both negatively stained with 2% (w/v) uranyl acetate. **(f)** Soluble fraction of peptide 12Bpro2 and 12Bscr2 (10 µM) determined after ultracentrifugation (250,000g for 30 min), measured over time. Mean values ± SD is shown (n = 3 independent experiments). **(g-h)** Dose-dependent effect of peptide 12Bpro2 (g) and 12Bscr2 (h) on area covered by plaques. Data is normalized to buffer-treated cells and the mean values ± SD is shown (n = 3 independent experiments).

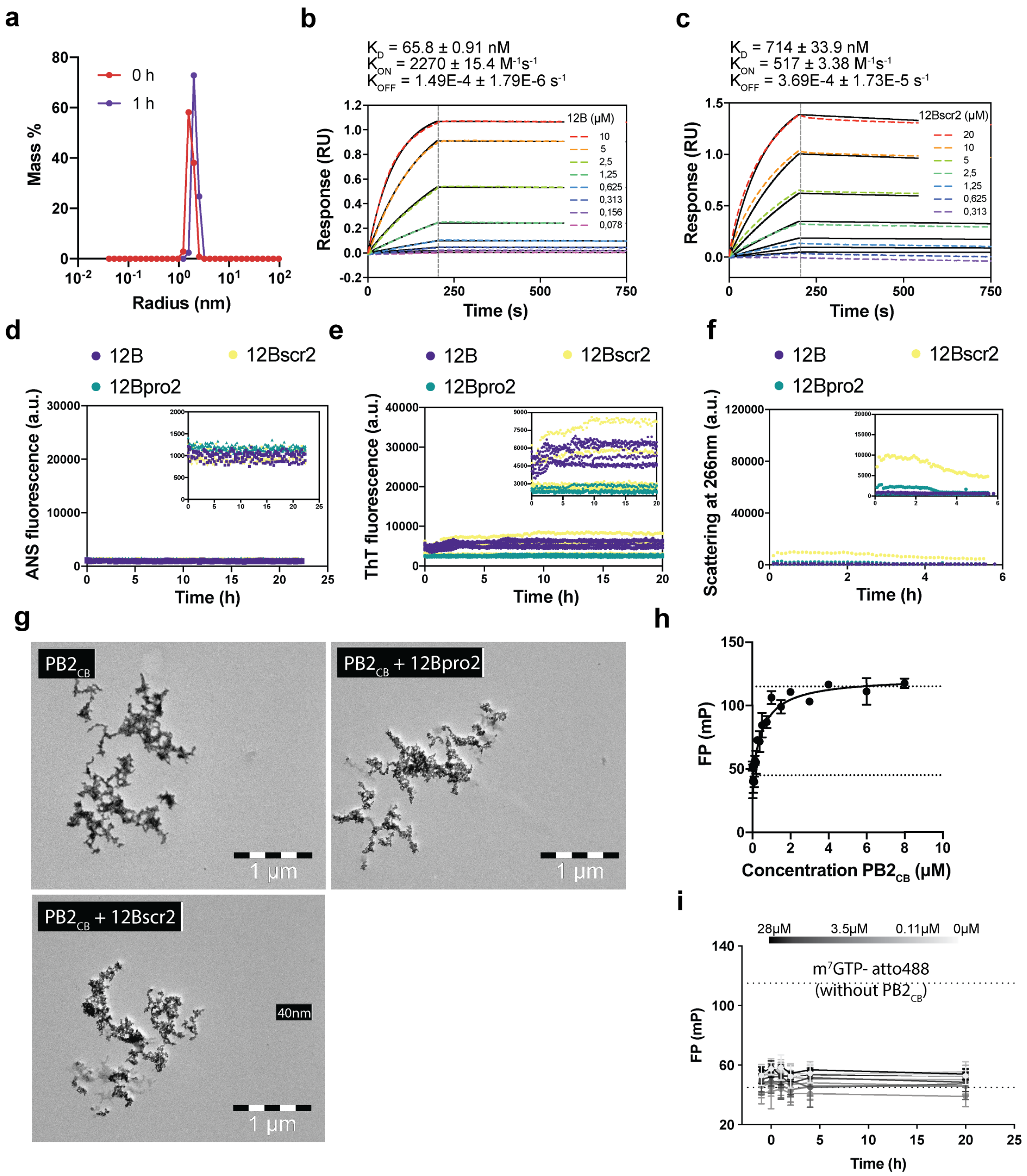

Supplementary Figure 4 – Mechanistic insights into the amyloid-viral protein interactions and controls in vitro. **(a)** Hydrodynamic radius (R_H) distribution calculated from the regularization fit of DLS data of freshly purified PB2$_{CB}$ (red) and after 1 hour incubation (purple) at room temperature. **(b-c)** Biolayer interferometry of different concentrations of peptide binding to his-tagged, immobilized PB2$_{CB}$. Association and dissociation were monitored for 200s and 550s, respectively. All kinetic constants were calculated from a global fitting, where a 1-1 binding model fitted best the experimental data. **(d-f)** ANS fluorescence (480 nm), Th-T fluorescence (485 nm) and light scattering (266 nm) of peptides (3.2 µM) alone over time. Graphs are scaled identical to Fig. 3b-3d and inserts correspond to the same data plotted at reduced scale on the Y axis. **(g)** TEM images of PB2$_{CB}$ (32 µM) without and with peptides (3.2 µM) after 20 hours incubation. **(h)** Binding of m^{7}GTP-atto488 (20 nM) by PB2$_{CB}$ at different concentrations as measured by FP. The dashed lines represent maximal binding of m^{7}GTP-atto488 to PB2$_{CB}$ (upper dashed line) and free m^{7}GTP-atto488 (lower dashed line). All

data points represent mean values ± SD (n = 4 independent experiments). **(i)** FP of m^{7}GTP-atto488 measured in presence of different concentrations of peptide 12B alone (no PB2$_{CB}$) as a function of time (grey scale for peptide concentration). The dashed lines represent maximal binding of m^{7}GTP-atto488 to PB2$_{CB}$ (upper dashed line) and unbound m^{7}GTP-atto488 (lower dashed line). All data points represent mean values ± SD (n = 4 independent experiments). A two-fold serial dilution of peptide 12B is used, starting from 28 µM (color gradient scale), and black dots represent peptide buffer only.

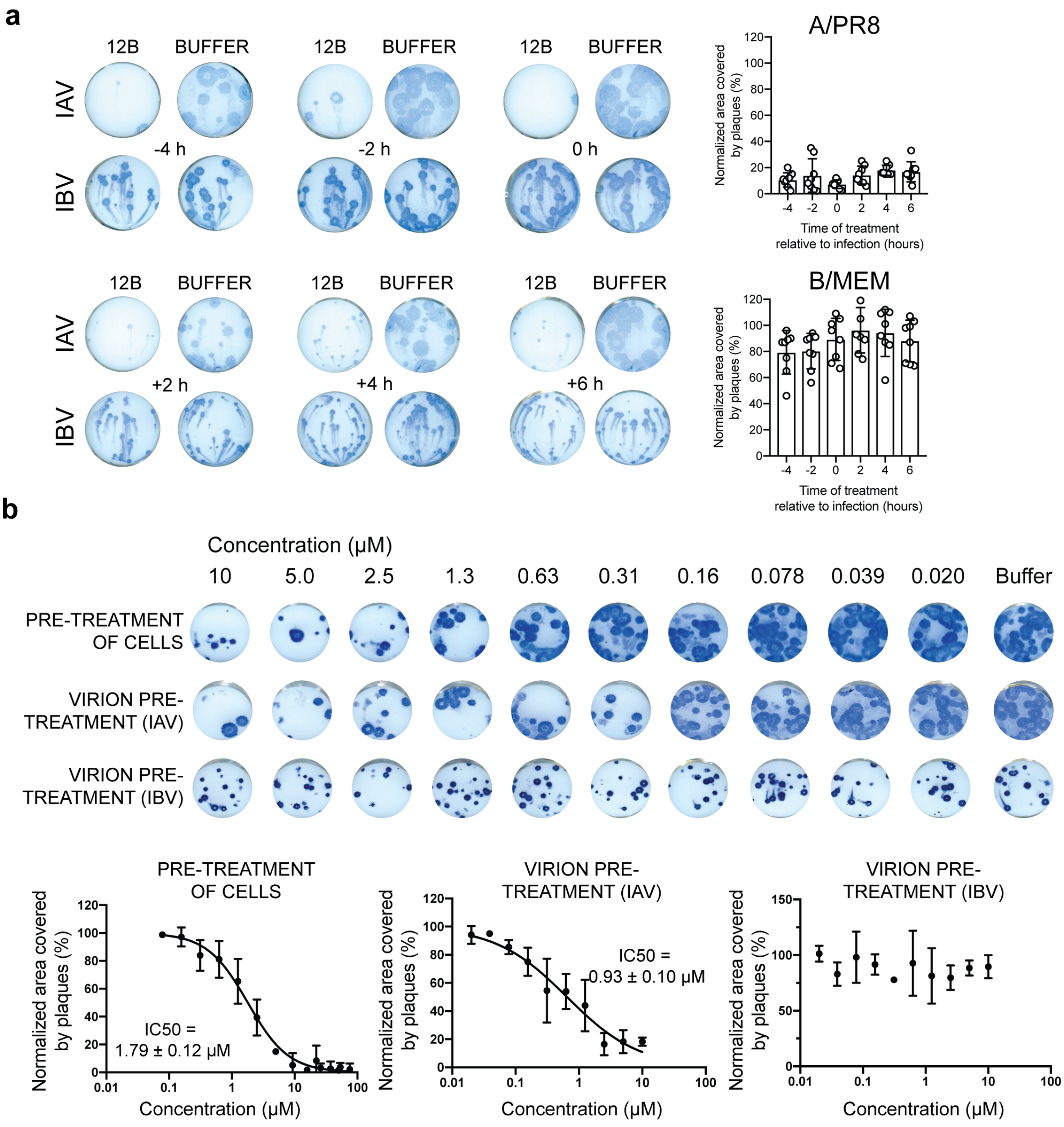

Supplementary Figure 5 – Amyloid peptide 12B does not interfere with viral entry of influenza A and influenza B. **(a)** Area covered by plaques in a plaque-size reduction assay of A/PR8- or B/Mem-infected MDCK cells treated with buffer or 10 µM peptide 12B at different time points relative to infection. Images are shown and graphs represent normalized plaque area relative to medium-treated cells. Mean values ± SD is shown (n = 8 from 3 independent experiments, statistics: one-way ANOVA with multiple comparison, p-value = 0.0605 (for IAV) and 0.226 (for IBV)). **(b)** Area covered by plaques in a plaque-size reduction assay of MDCK cells infected with A/PR8 or B/Mem. The images in the first row represent the typical assay in which MDCK cells are pretreated with peptide before virion addition. The images in

the second and third row represent pretreatment of virion particles before MDCK infection. The graphs represent the concentration dependent effect of peptide 12B normalized to medium-treated conditions. Mean values ± SD is shown (n = 3 independent experiments).

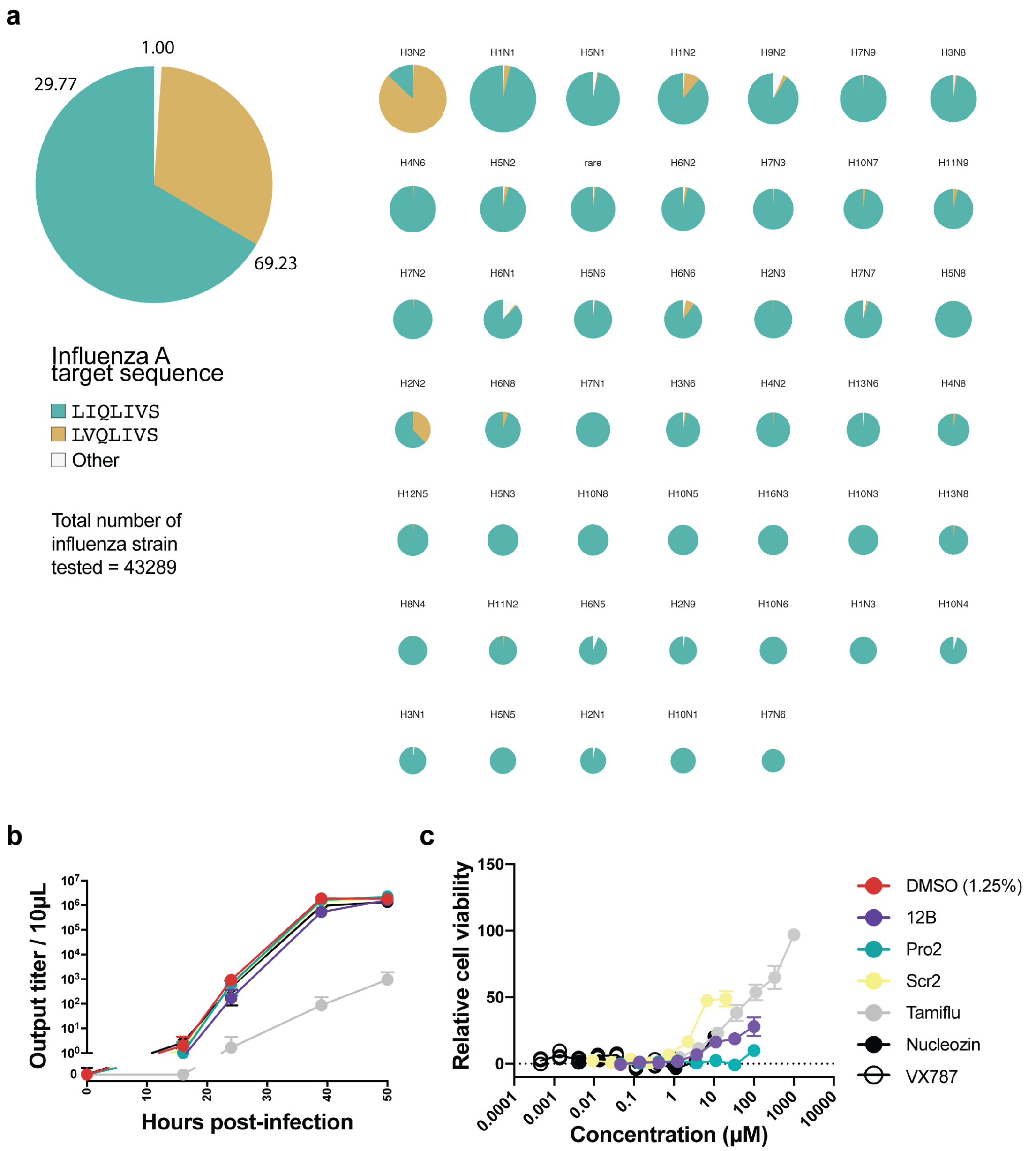

Supplementary Figure 6 – The PB2 target sequence is highly conserved in influenza A strains. **(a)** Analysis of the different polymorphisms of the target APR sequence in the alignment of 43.289 PB2 sequences from different influenza A strains (downloaded from the Influenza Database https://www.fludb.org/). **(b)** Multicycle replication of influenza B/Mem in MDCK cells. Cells were infected, supernatants were collected and checked for amount of virus at different time points post infection. Data represent mean values of virus titer per 10 µL supernatant ± SD (n = 4 independent experiments, statistics: one-way ANOVA with multiple comparison: DMSO-12B comparison p-value = 0.899). **(c)** Cytopathic effects of influenza B/Mem replication on MCDK cells. Cells were infected and treated with 1.25% DMSO, 10 µM peptide, 100 µM Tamiflu, 10 µM Nucleozin or 1 µM VX-787. After 72 hours, cytopathic effects were quantified by assaying cell viability. Data are normalized to non-infected (100% viability) and DMSO-treated, infected cells (0% viability) and shown as mean values ± SD (n = 4 independent experiments).

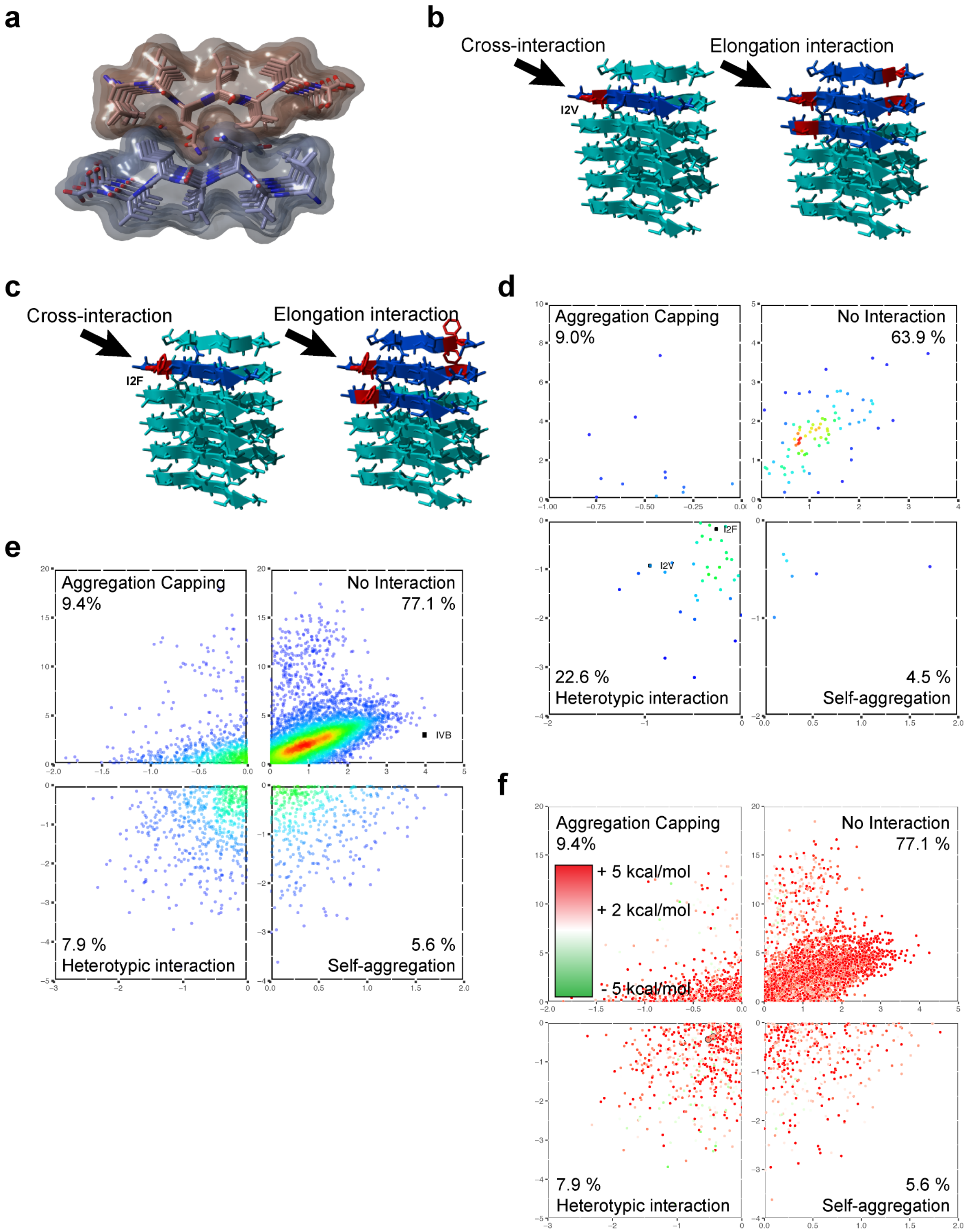

Supplementary Figure 7 – Calculating cross-aggregation specificity for the PB2 APR versus influenza A and B peptide segments. More information in Supplementary Note 2. **(a)** Structural representation of the generated LIQLIVS topology model (templated against PDB structure: 3HYD). **(b-c)** Structural representation of the calculated cross-interaction (left) and elongation (right) energies used to identify cross-specificity of the LIQLIVS aggregation prone peptide. The wild type APR, LIQLIVS, is shown in cyan and the variant sequence is shown in blue. Two different variants are shown here as an illustration: the I2V variant (b) and the I2F variant (c), and the mutated amino acid is shown in red. **(d-e)** Cross-specificity maps of (d)

single or (e) double mutations for the LIQLIVS model structure. The I2V and the I2F variant are visualized and are predicted to interact with the wild type APR. The color scale represent density of the spots (more dense from blue to red). **(f)** Identical data points as (e) with a color coding according to the calculated (FoldX) change in stability by inserting a specific double mutation.

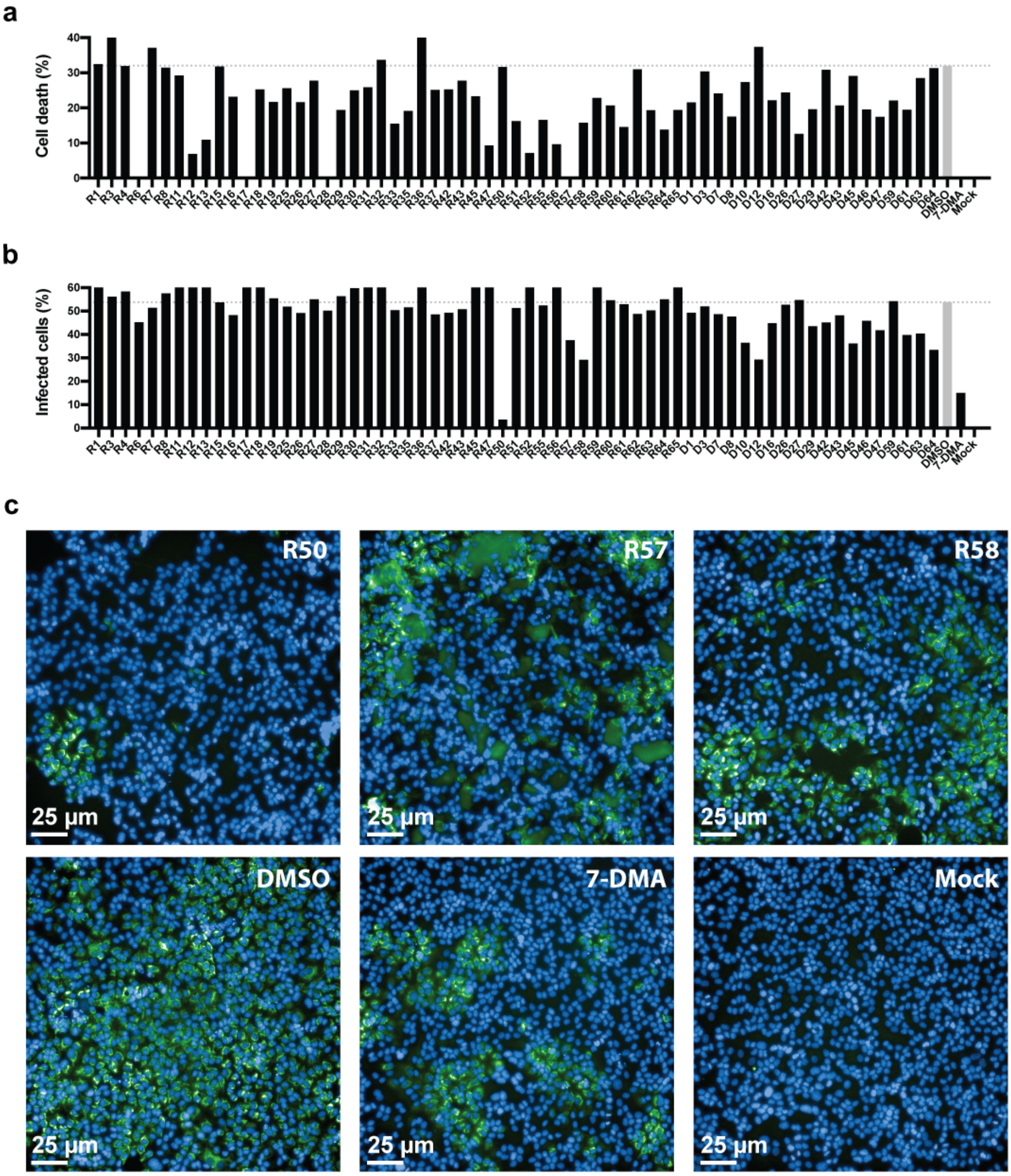

Supplementary Figure 8 – Screening of multiple APR-based peptides for inhibitory effects on ZIKV replication (MOI = 0.1). **(a)** Quantification of cell death following 48 hours of ZIKV infection (MOI = 0.1), with or without treatment, two hours prior to infection. Data is normalized to non-infected (mock), DMSO-treated cells. Grey dotted line represents cell death in DMSO-treated, infected cells. Data represent one replicate per condition. **(b)** Fraction of

infected cells following 48 hours of ZIKV infection (MOI = 0.1), with or without treatment two hours prior to infection. Grey dotted line represents fraction of infected cells in DMSO-treated, infected cells. Data represent one replicate per condition. **(c)** Representative images of the best performing peptides and all controls. Blue = DAPI; green = anti-Flavivirus antibody. Peptides and 7-DMA are dissolved in DMSO and used at a concentration of 20 μM and 10 μM, respectively. Figures represent one replicate per condition.

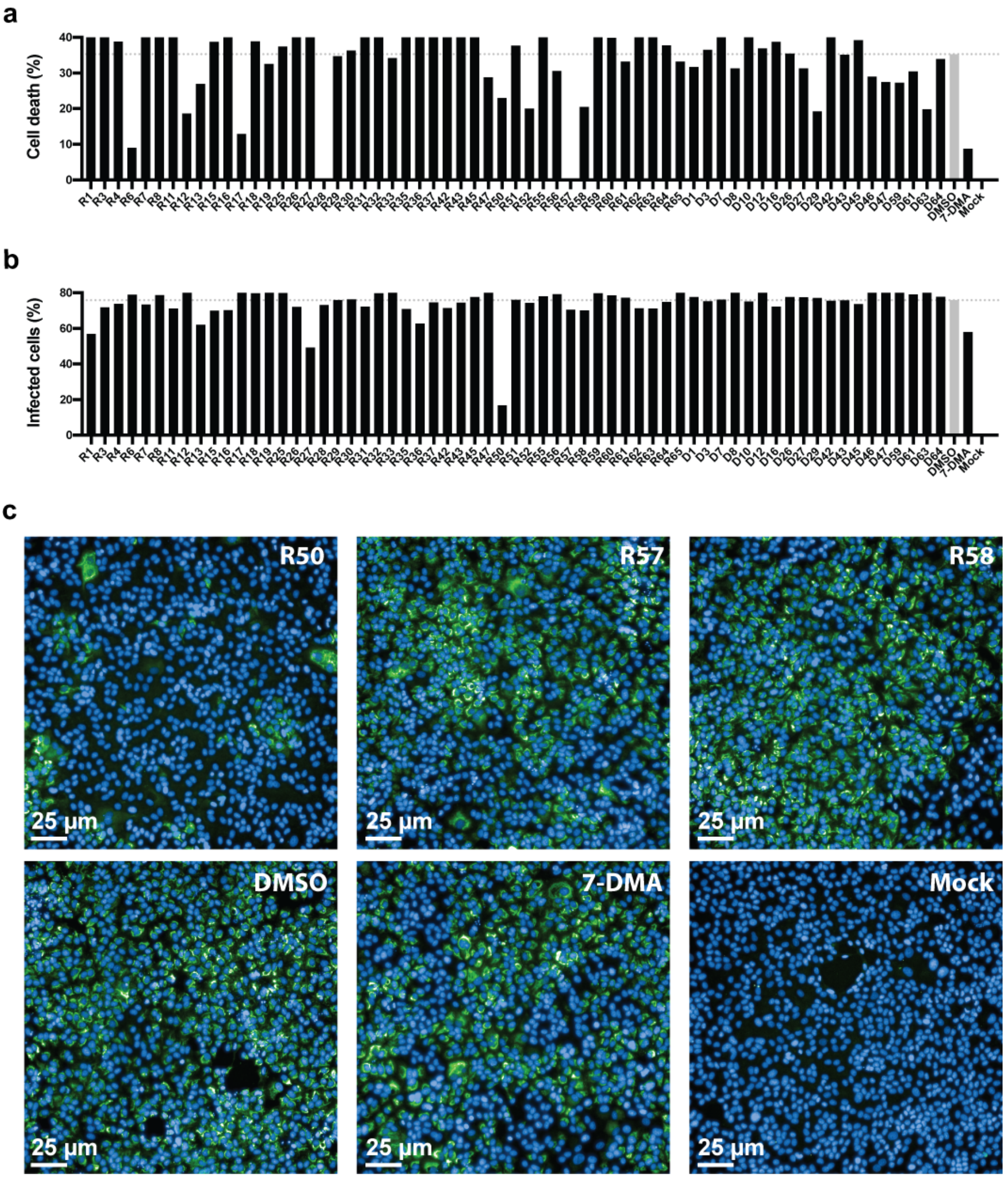

Supplementary Figure 9 – Screening of multiple APR-based peptides for inhibitory effects on ZIKV replication (MOI = 0.5). **(a)** Quantification of cell death following 48 hours of ZIKV infection (MOI = 0.5), with or without treatment, two hours prior to infection. Data is normalized to non-infected (mock), DMSO-treated cells. Grey dotted line represents cell death in DMSO-treated, infected cells. Data represent one replicate per condition. **(b)** Fraction of

infected cells following 48 hours of ZIKV infection (MOI = 0.5), with or without treatment two hours prior to infection. Grey dotted line represents fraction of infected cells in DMSO-treated, infected cells. Data represent one replicate per condition. **(c)** Representative images of the best performing peptides and all controls. Blue = DAPI; green = anti-Flavivirus antibody. Peptides and 7-DMA are dissolved in DMSO and used at a concentration of 20 µM and 10 µM, respectively. Figures represent one replicate per condition.

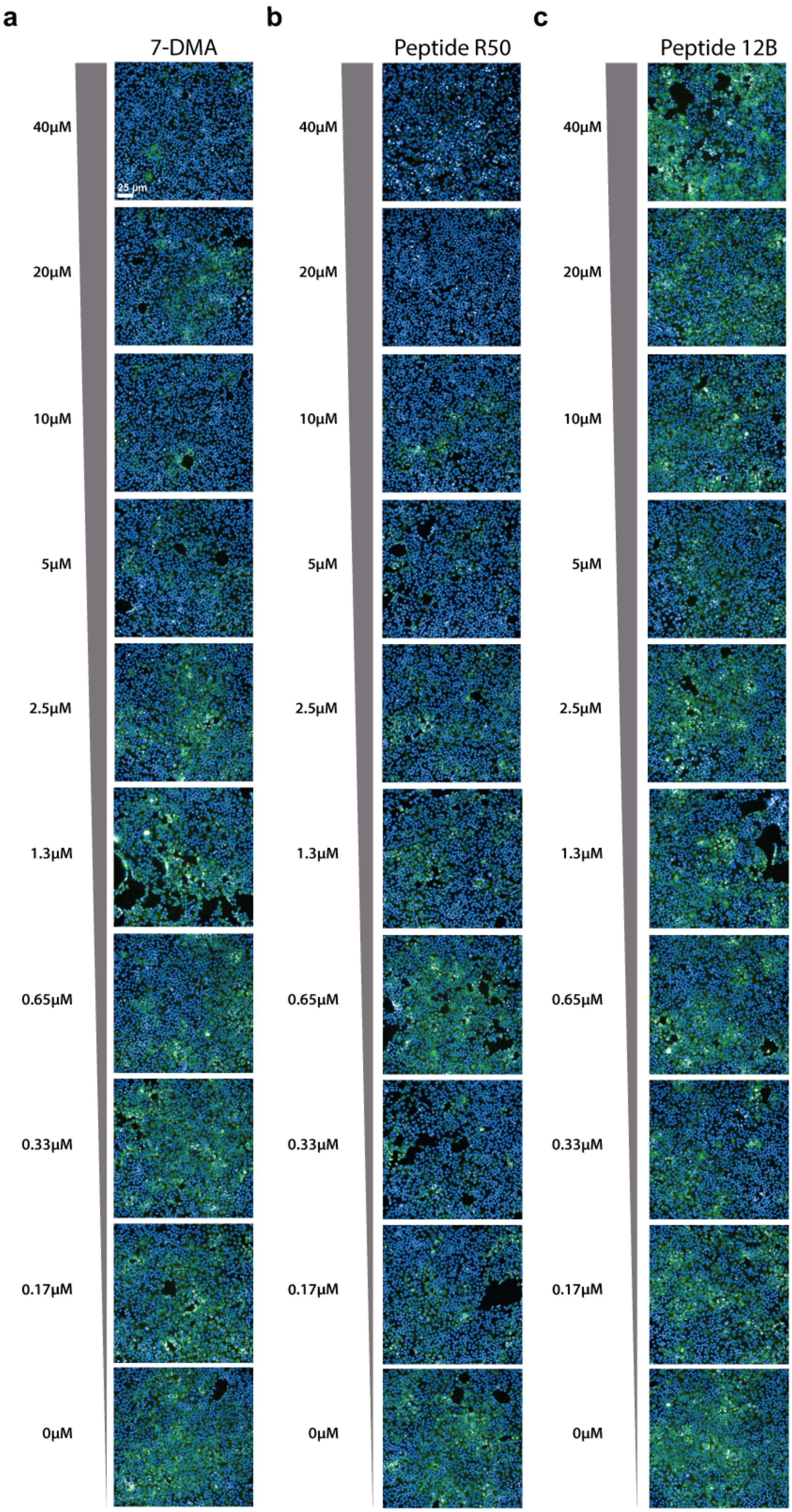

a
7-DMA
b
Peptide R50
c
Peptide 12B
40μM
20μM
10μM
5μM
2.5μM
1.3μM
0.65μM
0.33μM
0.17μM
0μM
25 μm

Supplementary Figure 10 – Peptide R50 specifically interferes with ZIKV replication in a concentration dependent manner. **(a-c)** Representative images of the dose-response effect of 7-DMA (**a**), peptide R50 (**b**) and peptide 12B (**c**) on the replication of ZIKV in Vero E6 cells (Figure 6). Blue = DAPI; green = anti-Flavivirus antibody. For all experiments, n = 3 independent experiments, representative images are shown. Scale bar is shown on the first image and all images are taken at the same magnification.

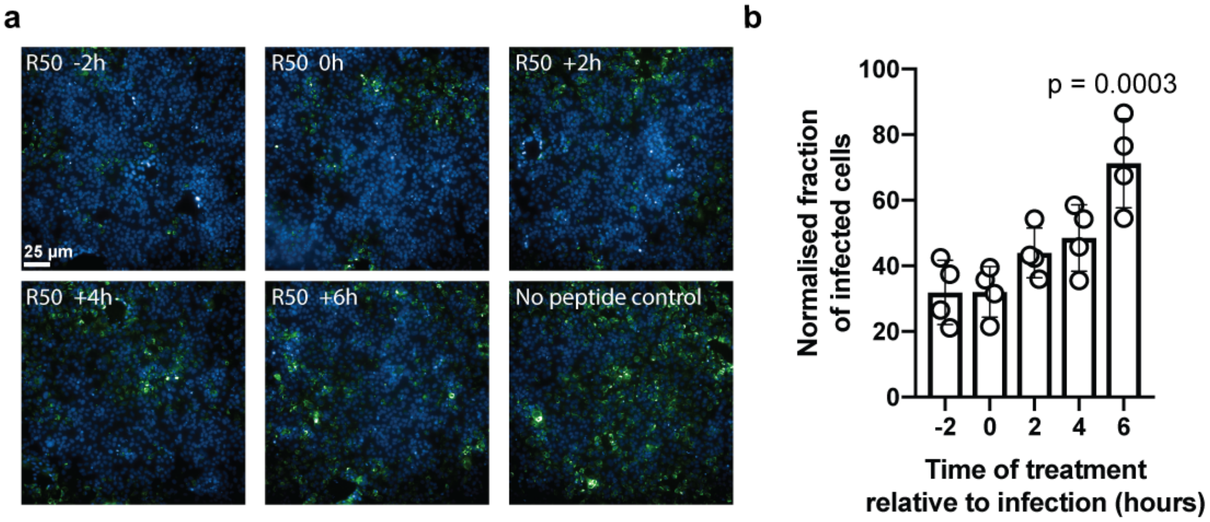

Supplementary Figure 11 – Time of addition assay for peptide R50 relative to ZIKV infection. **(a)** Representative images of Vero E6 cells non-treated and treated with peptide R50 at different time points relative to ZIKV infection (n = 4 independent experiments, representative images are shown). Scale bar is shown on the first image and all images are taken at the same magnification. **(b)** Quantification of the fraction of ZIKV-infected cells following 48 hours of infection with a MOI of 0.1 and treatment with peptide R50. Data is normalized to DMSO-treated, infected cells (100% infected cells) and mean values ± SD is shown (n = 4 independent experiments, statistics: ANOVA with multiple comparison). Only the final time points (6 hours) is significantly different than the other time points (p-value = 0.0003).

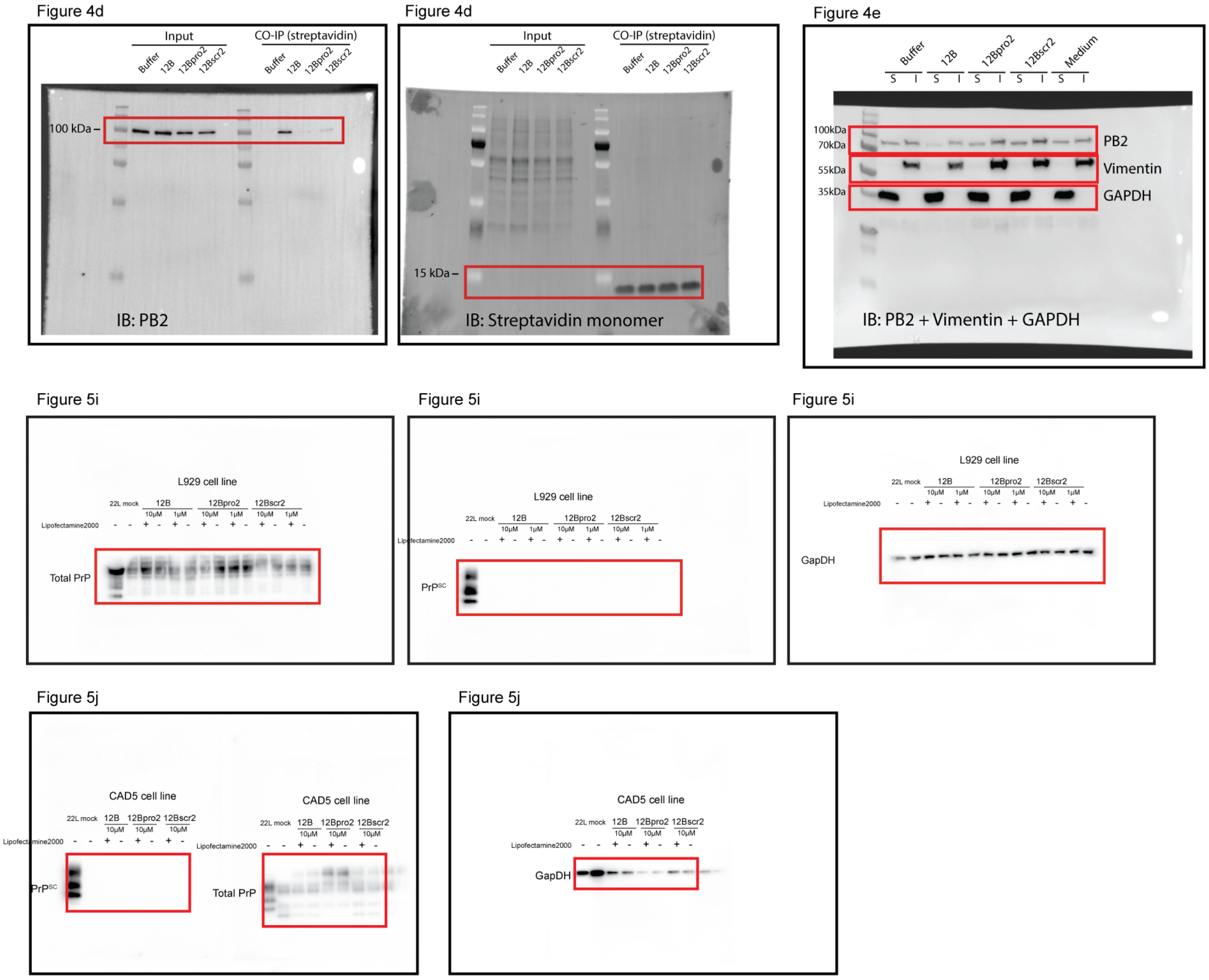

Supplementary Figure 12 – A representative image of the full blots represented in Figure 4 and Figure 5.

Peptide ID	Peptide sequence	Peptide ID	Peptide sequence
1	RNIAGWLLRPPRNIAGWLLR	19	RTTSVILTRPPRTTSVILTR
2	RSWSYIVRPPRSWSYIVR	20	RAVGVLIGRPPRAVGVLIGR
3	RNYYWTLLRPPRNYYWTLLR	21	RLMVAYMLRPPRLMVAYMLR
4	RMYAFALSRPPRMYAFALSR	22	RMALQLFIRPPRMALQLFIR
5	RTYVLSIIRPPRTYVLSIIR	23	RVAFGLVCRPPRVAFGLVCR
6	RWLTIGISRPPRWLTIGISR	24	RVYINTALRPPRVYINTALR
7	RNGAVAVLRPPRNGAVAVLR	25	RLLIVQALRPPRLLIVQALR
8	RVHIYYLRPPRVHIYYLR	26	RSIGVTVIRPPRSIGVTVIR
9	RNLYGFIIRPPRNLYGFIIR	27	RFGAIAGFRPPRFGAIAGFR
10	RLAVTWWNRPPRLAVTWWNR	28	RIIVAMVFRPPRIIVAMVFR
11	RQSLIIAARPPRQSLIIAAR	29	RFLTQGALRPPRFLTQGALR
12	RLIQLIVSRPPRLIQLIVSR	30	RTMAWTVVRPPRTMAWTVVR
13	RGFLILGRPPRGFLILGR	31	RFLAMITYRPPRFLAMITYR
14	RGFVYFVRPPRGFVYFVR	32	RVLVNTYQRPPRVLVNTYQR
15	RVLVLWGIRPPRVLVLWGIR	33	RNLLVLLCRPPRNLLVLLCR
16	RAYVSVVTRPPRAYVSVVTR	34	RILGFVFTLRPPRILGFVFTLR
17	RLVWMACHRPPRLVWMACHR	35	RWVLLNASWRPPRWVLLNASWR
18	RTTIMAAFRPPRTTIMAAFR	36	RGVSILNLRPPRGVSILNLR

Supplementary Table 1 – Peptides designed based on APRs identified in the influenza A/PR8 proteome. Only APRs shorter then 16 amino were selected and split into shorter 7- or 8-amino acid fragments to fit in our tandem design.

Peptide ID	Sequence
12A	WDLIQLIVSDPPDLIQLIVSD
12B	WDLIQLIVSDGSDLIQLIVSD
12C	WDLIQLHVSDPPDLIQLIVSD
12D	WDLIQLHVSDGSDLIQLIVSD
12E	WRLIQLIVSRPPRLIQLIVSR
12F	WRLIQLIVSRGSRLIQLIVSR
12G	WRLIQLHVSRPPRLIQLIVSR
12H	WRLIQLHVSRGSRLIQLIVSR

Supplementary Table 2– Variants of peptide 12 used in this study.

Peptide ID	Sequence
12Bpro2	WDLPQLPVSDGSDLPQLPVSD
12Bscr2	WDISQVLLIDGSDISQVLLID

Supplementary Table 3 – Control peptides (based on peptide 12B) used in this study.

Peptide ID	Peptide sequence	Peptide ID	Peptide sequence
R1	RAYYMYLRGSRAYYMYLR	R52	RLIVSYVVRGSRLIVSYVVR
R3	RLYYLTMRGSRLYYLTMR	R55	RISSAVLLRGSRISSAVLLR
R4	RTVVVLGRGSRTVVVLGR	R56	RAIWYMWLRGSRAIWYMWLR
R6	RIMLLSLRGSRIMLLSLR	R57	RLMVLINGRGSRLMVLINGR
R7	RLIASLYRGSRLIASLYR	R58	RALAWLAIRGSRALAWLAIR
R8	RALITAARGSRALITAAR	R59	RVWLAYQVRGSRVWLAYQVR
R11	RTLALAVIRGSRTLALAVIR	R60	RYQVASAGRGSRYQVASAGR
R12	RMWQLLYFRGSRMWQLLYFR	R61	RSAGITYTRGSRSAGITYTR
R13	RSYIVIGVRGSRSYIVIGVR	R62	RVLTAVGLRGSRVLTAVGLR
R15	RAAIFMTARGSRAAIFMTAR	R63	RALAGGFARGSRALAGGFAR
R16	RVVTGVTGRGSRVVTGVTGR	R64	RGFGMVTLRGSRGFGMVTLR
R17	RVLAILAFRGSRVLAILAFR	R65	RSAWLMWLRGSRSAWLMWLR
R18	RSTWVVYGRGSRSTWVVYGR	D1	DAYYMYLDGSDAYYMYLD
R19	RTAAFTFTRGSRTAAFTFTR	D3	DLYYLTMDGSDLYYLTMD
R25	RILMGATFRGSRILMGATFR	D7	DLIASLYDGSDLIASLYD
R26	RVAHLALVRGSRVAHLALVR	D8	DALITAADGSDALITAAD
R27	RHLALVAARGSRHLALVAAR	D10	DAVIGTAVDGSDAVIGTAVD
R28	RFVMALGLRGSRFVMALGLR	D12	DMWQLLYFDGSDMWQLLYFD
R29	RMALGLTARGSRMALGLTAR	D16	DVVTGVTGDGSDVVTGVTGD
R30	RINVVGLLRGSRINVVGLLR	D26	DVAHLALVDGSDVAHLALVD
R31	RVVGLLLLRGSRVVGLLLLR	D27	DHLALVAADGSDHLALVAAD
R32	RFAAGAWYRGSRFAAGAWYR	D29	DMALGLTADGSDMALGLTAD
R33	RAGAWYVYRGSRAGAWYVYR	D42	DYLAGASLDGSDYLAGASLD
R35	RNIVSSWLRGSRNIVSSWLR	D43	DAGASLIYDGSDAGASLIYD
R36	RMLLALASRGSRMLLALASR	D45	DIGIIGLLDGSDIGIIGLLD
R37	RLALASSLRGSRLALASSLR	D46	DLLTTAMADGSDLLTTAMAD
R42	RYLAGASLRGSRYLAGASLR	D47	DSAWAIYADGSDSAWAIYAD
R43	RAGASLIYRGSRAGASLIYR	D59	DVWLAYQVDGSDVWLAYQVD
R45	RIGIIGLLRGSRIGIIGLLR	D61	DSAGITYTDGSDSAGITYTD
R47	RSAWAIYARGSRSAWAIYAR	D63	DALAGGFADGSDALAGGFAD
R50	RVAIAWLLRGSRVAIAWLLR	D64	DGFGMVTLDGSDGFGMVTLD
R51	RMAAVGLLRGSRMAAVGLLR		

Supplementary Table 4 – Peptides designed based on APRs identified in the Zika virus (ZIKV) proteome (African strain MR766). Only APRs shorter then 16 amino where selected and split into shorter 7-amino acid fragments to fit in our tandem design. Both positively charged (arginine, R) as well as negatively charged (aspartate, D) gatekeeper residues were used in this design.

Supplementary Methods

Meta-analysis

The proteomes of all human-infecting viruses were downloaded from UniProt. A sliding window of six amino acids was used to cover the full primary sequence of all amyloids that are known to interact with human viruses. Homologues of these hexamers were identified in the proteomes of all human viruses by only allowing one amino acid mismatch. The number of times that each hexamer was found in a viral proteome was plotted against the primary sequence of the amyloid. Amyloid APRs were detected with TANGO as described above.

Peptides

Peptides were synthesized by solid phase peptide synthesis. An Intavis Multipep RSi synthesis robot was used for all peptides that were synthetized in-house. Of note, the maximum length of our peptides is ~20 amino acids, since the synthesis efficiency of our synthetic amyloids significantly drops beyond 20 amino acids. Based on this limitation, the tandem design restricts APR length to seven amino acids. After synthesis, crude peptides were stored as dry ether precipitates at –20°C. Crude peptides were only used in the initial screening assays (10 μM peptide was used, assuming 100% synthesis efficiency). Stock solutions of each peptide were either prepared in 100% DMSO (only for initial screening assays) or following the optimized protocol: peptides were dissolved in 1 M ammonium hydroxide, allowed to dissolve for ~5 minutes, and dried in 1.0 ml glass vials with a N_2 stream to form a peptide film. This film was dissolved in buffer containing 50 mM Tris (pH 8.0) and 20 mM guanidine thiocyanate. The concentration of the peptide stocks was determined by absorbance at 280 nm using the calculated molar extinction coefficient $\varepsilon = 5690\ M^{-1}cm^{-1}$. Pure peptides (>90%) were purchased from Genscript. All peptides were N-terminally acetylated and C-terminally amidated.

Antiviral activity screen

MDCK cells were seeded in complete DMEM medium in 24-well format (160,000 cells per well) and allowed to attach overnight. Cells were washed with serum-free DMEM medium and infected with virus (A/PR8 or A/NIBRG-14, both MOI 1) and treated with 10 μM peptide (assuming 100% synthesis efficiency). Sixteen hours later, the amount of newly produced virus in the supernatant was determined by TCID50 titration. For this, a 1:10 dilution series of output virus was used to infect MDCK cells in serum-free DMEM medium supplemented with 2 μg/mL TPCK-treated trypsin. After 6 days, endpoint titers were determined by the method of Reed and Muench[1] upon agglutination of supernatants using a 1% chicken red blood cell suspension.

Multicycle infection assay

MDCK cells were seeded in complete DMEM medium in 24-well format (160,000 cells per well) and allowed to attach overnight. Cells were washed once with serum-free DMEM medium, treated with DMSO-solubilized peptide (10 μM), DMSO (1.25%), nucleozin (10μM) or Tamiflu (400μM), and infected with virus two hours later. Two hours after infection, inoculums were removed and fresh serum-free DMEM medium, supplemented with 2 μg/mL TPCK-treated Trypsin, was added. At the indicated time points after removal of inoculums, a small sample was taken from each well for titration by 96-well format plaque assay. For this, MDCK cells were seeded (25,000 cells per 96 well) and allowed to attach overnight. Cells were washed once with serum-free DMEM medium and incubated with a dilution series of virus samples. Plaque assay was performed as described before, with an incubation time of

24h, after which cells were fixed and plaques were visualized using convalescent serum and True-Blue staining as described before.

Protection against cytopathic effects of virus

Protection from virus-induced cytotoxicity was examined using the CellTiter-Blue Cell Viability Assay (Promega, USA). Briefly, MDCK cells were seeded (25,000 cells per 96 well) and allowed to attach overnight. Cells were washed once with serum-free DMEM medium and treated with a dilution series of peptides or antivirals. Two hours after treatments, a predefined amount of virus was added to each well, equivalent to the titer yielding 90% cytotoxicity in this assay. Control wells included no virus infection (100% viability), and virus-infected buffer- or medium-only treated (set to 0% viability). Two hours after infection, serum-free DMEM was added, supplemented with TPCK-treated Trypsin to reach a final concentration of 2 µg/mL (final volume in wells: 100 µL). Three days after infection, 20 µL of the CellTiter Blue reagent was added to each well and plates were incubated for an additional three hours. Fluorescence was measured at 590 nm, upon excitation at 560 nm, using the Infinite 200 Pro Micoplate Reader (Tecan, Austria).

Alignment

43.289 PB2 sequences originating from different influenza A strains were downloaded from the influenza database (https://www.fludb.org). The alignment of all sequences was performed with MAFFT (Multiple Alignment by Fast Fourier Transformation) and analysis of $L_{381}IQLIVS_{387}$ was done with MEGA7 (Molecular Evolutionary Genetics Analysis).

Protein purification

DNA encoding the cap-binding domain of PB2 of influenza A/PR8 (residues 318–483) and influenza B/Mem (residues 319–484) were each subcloned into a pET30a vector, with an N-terminal HIS-tag followed by the TEV cleavage site. The proteins were expressed in *E. coli* (strain DE3) by inducing with 0.5 M IPTG for 16 hours at 18 °C. Cells were harvested by centrifugation (15 minutes at 6,200 x g), resuspended in buffer (50mM Tris pH 8.0, 300mM NaCl, 5mM beta-mercaptoethanol) and lysed using a high-pressure homogenizer (EmulsiFlex C5, Avestin, Canada). The cell debris was removed by centrifugation (30 minutes, 39,000 x g) and the soluble lysate was loaded on a nickel affinity column (HisTrap Fast Flow column, 5 ml column volume, GE Healthcare Life Sciences). The protein was eluted with a linear gradient of imidazole from 20 mM to 400 mM over ten column volumes. The eluted protein was incubated overnight with His-tagged TEV protease at 4°C. A second nickel affinity chromatography was performed to separate the PB2 protein from the cleaved His-tag, the un-cleaved His-PB2 and the His-TEV proteins. Finally, a gel filtration step (SuperDex 75 column, GE healthcare) was performed right before every experiment to obtain a monodisperse solution of $PB2_{CB}$.

In vivo dose-escalation study

5 weeks old BALB/C female mice were injected intravenously (100 µL) via the tail caudal vein (i.v.) in each group with increased dose levels (0.5; 1; 2; 4; 8; 10mg/kg) respectively. The control mice were administered 100 µL of peptide buffer. The biological reactivity that was recorded included mortality, clinical signs such as piloerection, dyspnea, tremor and total body-weight gain, food consumption, and gross necropsy findings were recorded.

In vivo toxicity study

5 weeks old BALB/C female mice received daily injections (100 µL) via the tail caudal vein (i.v.) of 10 mg/kg peptide or buffer for 14 days. After 14 days of treatment, blood samples were

collected from each animal using a standard retro–orbital puncture and histopathology examination were done. To perform the histopathology, mice were first weighed and deeply anesthetized by Nembutal 50mg/kg. Subsequently, paraformaldehyde 4% and then PBS buffer was gently perfused into the heart ventricle with a 50mL-syringe till the tail and the four legs of the animal became stiff. The desired organs (heart, liver, spleen, kidney, bone marrow, brain, lung) were dissected and put into paraformaldehyde 4% as a fixative buffer. After one hour, organs were washed in PBS and moved into ethanol 70%, in order to dehydrate the tissues. All organs were trimmed into multiple cassettes and processed into paraffin-embedded tissue blocks. Each block had thin (~4 µm) sections cut on a microtome, mounted on glass slides, and stained with hematoxylin and eosin (H&E) to evaluate lesions, morphological abnormality, and cellular infiltration. Evaluation of the microscopic slides was performed using a Nikon Labophot-2 microscope, and high-resolution (16x to 1000x magnification) digital images were captured by a Nikon D5000 digital microscopic camera.

Synthesis of ^{68}Ga-labelled 12B peptide

For radiolabeling, peptide 12B was conjugated to a PEG_2 spacer followed by coupling to 2,2'-(7-(1-carboxy-4-((2,5-dioxopyrrolidin-1-yl)oxy)-4-oxobutyl)-1,4,7-triazonane-1,4-diyl)diacetic acid (NODAGA-NHS, active ester of NODAGA) as a chelator for gallium-68. [^{68}Ga]Ga-NODAGA-PEG2-12B was produced using a SCINTOMICS GRP module in combination with a disposable GMP grade cassette system (ABX advanced biochemical compounds). All reagents were carefully selected to minimize presence of metal contaminants, e.g. iron. Gallium-68 was eluted in the form of [^{68}Ga][GaCl4]- from a commercially available IGG101 Pharmaceutical Grade Generator from Eckert & Ziegler (Berlin, Germany) using 0.1 M HCl. To concentrate gallium-68 and remove any germanium-68 in the generator eluate, the mixture was applied on a Chromafix PS-H+ column (Macherey-Nagel, Germany). ^{68}Ga was eluted with 1.5 ml of 5 M NaCl into the reactor vial containing 15-30 nmol of peptide. The radiolabeling mixture was heated for 6 min at 65 °C. After cooling, the reaction mixture was passed over a Sep-Pak C18 Light cartridge (Waters). The Sep-Pak C18 Light cartridge was pre-conditioned with ethanol (5 ml) and water-for-injection (5 ml). The radiolabeled peptides were eluted from the cartridge with 2 ml ethanol/water (75:25) and diluted with PBS (EtOH content below 10%). Finally, sterile filtration was performed using a vented 0.22 µm filter (Cathivex Merck Millipore).

Biodistribution studies

Healthy or influenza-infected mice were anesthetized with 2.5% isoflurane in O2 at a flow rate of 1 l/min and injected with 0.5-1.5 MBq of [^{68}Ga]Ga-NODAGA-PEG2-12B via a tail vein. Mice were euthanized by decapitation at different time points from 2 min to 2 h after injection and blood and organs of interest were collected and weighed. The radioactivity present in the tissues was measured in an automated gamma counter which contained a 3-inch NaI(Tl) well crystal linked to a multichannel analyzer (Wallac 1480 Wizard, Wallac, Turku, Finland). For quantification, counts were corrected for background, counter dead time and physical decay during counting. Tissue and organ uptake was calculated as percentage of injected dose (%ID, calculated as (counts per minute (cpm) in organ/total cpm recovered) x 100) and SUV (calculated as (radioactivity in cpm in organ/weight of organ in grams)/(total cpm recovered/body weight in grams)). For the calculation of the total radioactivity in blood, muscle and bone, the masses were considered to be 7%, 40% and 12% respectively of the total body mass. In figures displaying %ID, "urine" is %ID of excreted urine and the bladder combined to represent the total urinary elimination.

Immunofluorescence anti-ZIKV screen

Cells were plated in 96-well black viewplates (Greiner) and allowed to adhere overnight (10,000 cells per well). Peptides were dissolved in 100% DMSO and diluted 200-fold in assay medium prior to treatment. The viral polymerase inhibitor 7-deaza-2'-C-methyladenosine (7DMA) was included as a positive control[2]. Two hours after treatment, Vero E6 cells were infected with ZIKV (African strain MR766) at a multiplicity of infection (MOI) of 0.1 and allowed to incubate for 48 hours. Cells were fixed with 4% paraformaldehyde in PBS and immunostainings were performed with DAPI (nuclear staining) and an anti-flavivirus antibody targeting the envelope protein of flaviviruses (Anti-Flavivirus Group Antigen Antibody, clone D1-4G2-4-15 (Millipore)) as the primary antibody, followed by an alexa488 anti-mouse secondary antibody. The number of cells was determined by counting the nuclei based on the DAPI staining. The fraction of infected cells was determined by counting the number of cells positive for anti-flavivirus staining. Read out was performed using a high-content imager (ArrayScan XTI High Content Analysis Reader, Thermo Scientific).

Supplementary Table 5

REAGENT or RESOURCE	SOURCE	IDENTIFIER
**Antibodies**		
Goat anti-RNP	Bei Resources	Cat# NR-3133
Anti-goat-HRP	Santa Cruz Biotechnology	Cat# SC-2020
Anti-mouse-HRP	ThermoFisher	Cat# 62-6520
Anti-FLAG antibody	Cell Signaling	Cat# D6W5
Anti-vimentin antibody	Santa Cruz Biotechnology	Cat# SC-V9
Anti-GAPDH antibody	Santa Cruz Biotechnology	Cat# SC-6C5
Anti-GAPDH antibody	Abcam	Cat# ab8245
Anti-rabbit antibody-AlexaFluor594	ThermoFisher	Cat# R37117
Anti-Flavivirus Group Antigen Antibody, clone D1-4G2-4-15	Millipore	Cat# MAB10216
**Bacterial and Virus Strains**		
A/Puerto Rico/8/34 (H1N1)	Prof Dr John Skehel, Mt Sinai School of Medicine, NY	N/A
A/NIBRG14	National Institute of Biologicals	N/A
A/Swine Ontario/42729A/2001 (H3N3)	Dr. Carman, Guelph University, Ontario	N/A
A/Udorn/307/72 (H3N2)	Prof Peter Staeheli, Germany	N/A
A/Chicken/Nanchang/3-120/2001 (H3N2)	Ashley Webb, St Jude Children's Research hospital, Memphis	N/A
A/Duck/Ukraine/1/63 (H3N8)	Prof Dr John Skehel, Mt Sinai School of Medicine, NY	N/A
A/WSN-FLAG (H1N1)	Nadia Naffakh, Institute Pasteur, Lille	N/A
B/Memphis/12/97 (Yamagata-lineage)	Jonathan McCullers, Department of Infectious Diseases, St. Jude Children's Research Hospital, Memphis	N/A
B/Wisconsin/01/2010 (Yamagata-lineage)	Vicki Gregory, Mill Hill, London	N/A
B/Brisbane/60/2008 (Victoria-lineage)	Vicki Gregory, Mill Hill, London	N/A
BL21(DE3) Competent E. coli	NEB	Cat# C2527I
Zika virus African strain MR766	Prof. Johan Neyts	N/A
**Biological Samples**		

**Chemicals, Peptides, and Recombinant Proteins**		
Peptides (custom made)	Genscript	N/A
Aβ42	rPeptide	Cat# A-1163-1
Aβ42-HiLyte647	AnaSpec	Cat# AS-64161
amylin	EuroGenTec	Cat# AS-60804
EDA-m7GTP - ATTO-488	Jena bioscience	Cat# NU-824-488
oseltamivir phosphate	Roche	N/A
Nucleozin	Sigma	Cat# N2790<br>CAS Number 341001-38-5
Dynabeads™ M-280 Streptavidin	ThermoFisher Scientific	Cat# 11205D
ProLong Gold Antifade with DAPI	ThermoFisher scientific	Cat# P36935
**Critical Commercial Assays**		
CellTiter-Blue Cell Viability Assay	Promega	Cat# G8080
**Deposited Data**		
**Experimental Models: Cell Lines**		
MDCK	ATCC	Cat# CCL-34
HEK 293	ATCC	Cat# CRL-1573
Mouse fibroblast cell line L929	Sigma-Aldrich	Cat# 85103115
CAD5 cells	gift from Corinne Lasmezas	N/A
**Experimental Models: Organisms/Strains**		
BALB/c wild type mice	Charles River	N/A
**Oligonucleotides**		
**Recombinant DNA**		
pET30a-IAV-PB2CB-His	Switch Lab Stock	N/A
pET30a-IBV-PB2CB-His	Switch Lab Stock	N/A
**Software and Algorithms**		
Graphpad Prism (Version 7.0)	Graphpad software	http://www.graphpad.com/<br>RRID:SCR_002798
R-Studio	R	https://www.rstudio.com/<br>RRID:SCR_000432
MEGA 7 (Version 7.0.21)	MEGA	https://www.megasoftware.net/<br>RRID: SCR_000667
ImageJ (Version 1.51h)	NIH Image	https://imagej.nih.gov/ij/<br>RRID: SCR_003070
MAFFT (Version 7)	MAFFT	https://mafft.cbrc.jp/alignment/software/<br>RRID: SCR_011811
**Other**		
Octet Red96 System	ForteBio, Pall	N/A
ClarioStar plate reader	BMG labtech	N/A
PolarStar Optima plate reader	BMG labtech	N/A
DynaPro DLS plate reader	Wyatt	N/A

Bruker Tensor 27 infrared spectrophotometer	Bruker	N/A
JEM-1400 transmission electron microscope	Jeol	N/A
Monolith NT. Automated	NanoTemper	N/A
Optim1000	Unchained Labs	N/A

Supplementary Notes

Supplementary Note 1 – In vivo toxicity study.

To evaluate the tolerance for our synthetic peptide, all major organs of the mice used in the toxicity study were prepared for staining with Hematoxylin and Eosin (H&E) and checked for lesions. All slides were transferred to, and evaluated by, a pathologist. Specimens were examined for the presence and extent of lesions, morphological abnormality, and cellular infiltration. The severity of microscopic lesions was graded subjectively according to the following scheme:

- **NSL**: No significant lesions, or normal morphology with no significant tissue effects. The assigned grade was **Grade 0**.
- **Minimal**: Corresponds to a microscopic change that may be barely noticeable or changes so minor, small, or infrequent as to warrant no more than the least assignable grade. This grade was used where ≤10% of the tissue type in the defined area was involved. The assigned grade was **Grade 1**.
- **Mild**: Corresponds to a microscopic change that was a noticeable but not a prominent feature of the tissue. For focal, multifocal, or diffusely distributed tissue effects, this grade was used where 11-20% of the tissue type in the defined area was involved. The assigned grade was **Grade 2**.
- **Moderate**: Corresponds to a microscopic change that was a prominent feature of the tissue. For focal, multifocal, or diffusely distributed tissue effects, this grade was used where 21-40% of the tissue type in the defined area was involved. The assigned grade was **Grade 3**.
- **Marked**: Corresponds to a microscopic change that was an overwhelming feature of the tissue. For focal, multifocal, or diffusely distributed tissue effects, this grade was used where 41-100% of the tissue type in the defined area was involved. The assigned grade was **Grade 4**.

In addition, a diagnostic code was added to describe the type of lesion observed: 4, Extramedullary hematopoiesis; 5, Glomerulonephritis, mesangioproliferative; 8, Lymphoreticular cell infiltration; 30, Inhalation pneumonia; 31, Hepatitis, necrotic, microfocal; 35, Hepatic glycogen accumulation; 36, Hepatic fatty change; 68, Hemorrhages; 69, Lymphoid infiltration; 70, Congestion; 71, Hepatocytomegaly and karyomegaly.

Pathologist summary: The mice in the two control groups (non-injected and buffer-injected) and the test article group (peptide treated) had minimal to mild lesions associated with the method of euthanasia, such as hypostatic congestion and multifocal pulmonary hemorrhages. The variable amount of hepatic glycogen accumulation in all experimental groups was related to the nutritional status prior to necropsy and not associated with the administration of peptides. Spontaneous lesions such as hepatic extramedullary hematopoiesis showed no association with the administration of the peptide, the buffer control or non-injection control groups. The amount of hepatic extramedullary hematopoiesis was very low in all of the experimental animals. There were some mild lesions in the peptide groups that separated them from the buffer control and non-injection control groups. These minimal to mild lesions represent a physiological response of the animals' reticuloendothelial system to remove the injected foreign proteinaceous material from the animal's body, and not a specific toxicity response. The lesions consisted of a minimal mesangioproliferative glomerulonephritis in the peptide treated mice. Also, the group that received the peptides had a slight irregular increase in lymphoreticular cells forming a minimal to mild mantel around the splenic lymphoid follicles. These two renal and splenic lesions are not expected to result in any significant clinical effects.

Overall the mice in this study were relatively free of spontaneous microscopic lesion that would confound the study.

Supplementary Note 2 – In silico mutational analysis of the PB2 target APR.
First, we modeled the amyloid steric zipper structure of our target APR, LIQLIVS, using a Cordax-based approach (http://cordax.switchlab.org/, **Suppl. Fig. 7a**). In short, modeling was performed by threading the LIQLIVS sequence against several hepta-peptide steric zipper structures available from PDB. Finally, the best energetically fitted model (lowest dG) was selected using FoldX stabilities. Based on this model we calculated the effect on the cross-interaction potential of all possible single- and double mutations with the wild type APR sequence. More specifically, for each of these mutations, we calculated the effect on the cross-interaction and elongation energies relative to the interaction energies of the wild type APR sequence (**Suppl. Fig. 7b-7c**). The cross-interaction energies refer to the energy calculation when a mutant APR stretch is added to a preformed aggregate of the wild type APR stretch (**Suppl. Fig. 7b-7c, left panels**). If the cross-interaction energy is negative, the mutant and wild type APR are predicted to cross-interact, resulting in a heterotypic interaction. If the cross-interaction energy is positive, heterotypic interactions are unlikely to occur according to our prediction. The elongation energies refer to the energy calculation when more mutant APR stretches are added (**Suppl. Fig. 7b-7c, right panels**). If this energy is negative, the mutant APR is predicted to continue the amyloid elongation reaction, but if not, the reaction is predicted to stop. These calculations yield the quadrant plots visualized in **Suppl. Fig. 7d-7e**. On these plots the x-axis represents the cross-interaction energy and the y-axis represents the elongation energy and every dot represent a single (**Suppl. Fig. 7d**) or double (**Suppl. Fig. 7e**) mutation. So, if a dot is shown on the right side of a quadrant plot, this mutant will not be able to form amyloid interactions with the wild type LIQLIVS sequence. If a dot is shown in the lower left quadrant, this mutant sequence can still co-aggregate with the wild type LIQLIVS sequence. As described in the main text, when used multiple influenza A strains across different subtypes (H1N1, H3N2, H3N3, H3N8, H5N1), amyloid peptide 12B retained activity against all the frequently occurring single polymorphisms of the target APR are represented ($_{381}$LIQLIVS$_{387}$ to $_{381}$L**V**QLIVS$_{387}$ or $_{381}$L**F**QLIVS$_{387}$, **Fig. 5a**). This is in agreement with our *in silico* analysis, as these single amino acid variants are expected to interact with the wild type APR; they are located in the lower left quadrant. The influenza B APR, which contains 6 mutations compared to the wild type sequence, is also visualized in the plot and is predicted to not interact with the wild type APR. Overall, 31.6% of all single mutant sequences are expected to interact with the wild type APR sequence, while this number drops to 17.3% for double mutant sequences. Next, we calculated the overall stability of the PB2 protein when these double mutations are inserted, using FoldX. **Suppl. Fig 7f** represent the same plot as **Suppl. Fig. 7e** but a color code is used to show the effect of the double mutants: a green dot represents a change in dG smaller then +2 kcal/mol, and a red dot represents a larger increase in dG. This analysis shows that most double mutants that still allow interaction with the wild type APR sequence have a large destabilizing effect on the full protein and compensating mutations would be necessary to allow this protein to be stable.

Supplementary References

1. Reed, l. j. & Muench, h. A simple method of estimating fifty per cent endpoints. American Journal of Epidemiology 27, 493–497 (1938).
2. Zmurko, J. et al. The Viral Polymerase Inhibitor 7-Deaza-2'-C-Methyladenosine Is a Potent Inhibitor of In Vitro Zika Virus Replication and Delays Disease Progression in a Robust Mouse Infection Model. PLoS Negl Trop Dis 10, e0004695 (2016).
