# Supplementary material for: Reverse engineering synthetic antiviral amyloids
Source: Nat Commun. 2020 Jun 5;11:2832. doi: 10.1038/s41467-020-16721-8 (PMC7275043; doi:10.1038/s41467-020-16721-8)
Supplement: Supplementary file 3 — Reporting Summary [file 41467_2020_16721_MOESM3_ESM.pdf]

nature research

Corresponding author(s): Frederic Rousseau

Last updated by author(s): Jan 31, 2020

Reporting Summary

Nature Research wishes to improve the reproducibility of the work that we publish. This form provides structure for consistency and transparency in reporting. For further information on Nature Research policies, see Authors & Referees and the Editorial Policy Checklist.

Statistics

For all statistical analyses, confirm that the following items are present in the figure legend, table legend, main text, or Methods section.

n/a	Confirmed	
☐	☒	The exact sample size (*n*) for each experimental group/condition, given as a discrete number and unit of measurement
☐	☒	A statement on whether measurements were taken from distinct samples or whether the same sample was measured repeatedly
☐	☒	The statistical test(s) used AND whether they are one- or two-sided<br>*Only common tests should be described solely by name; describe more complex techniques in the Methods section.*
☒	☐	A description of all covariates tested
☐	☒	A description of any assumptions or corrections, such as tests of normality and adjustment for multiple comparisons
☐	☒	A full description of the statistical parameters including central tendency (e.g. means) or other basic estimates (e.g. regression coefficient) AND variation (e.g. standard deviation) or associated estimates of uncertainty (e.g. confidence intervals)
☐	☒	For null hypothesis testing, the test statistic (e.g. *F*, *t*, *r*) with confidence intervals, effect sizes, degrees of freedom and *P* value noted<br>*Give P values as exact values whenever suitable.*
☒	☐	For Bayesian analysis, information on the choice of priors and Markov chain Monte Carlo settings
☒	☐	For hierarchical and complex designs, identification of the appropriate level for tests and full reporting of outcomes
☒	☐	Estimates of effect sizes (e.g. Cohen's *d*, Pearson's *r*), indicating how they were calculated

Our web collection on statistics for biologists contains articles on many of the points above.

Software and code

Policy information about availability of computer code

Data collection	PerkinElmer Harmony (high content operetta imaging), Leica Application suite (confocal imaging). Additional data collection is described in the Methods section.
Data analysis	Graphpad Prism 8.0.1 (biological data analysis), R-studio 1.1.456 (biological data analysis), MEGA 7.0.21 (sequence alignment tool), ImageJ 1.51h (biological data analysis), YASARA 16.4.6 (3D protein structure visualization), Image Lab 5.2.1 (Western blot analysis), MAFFT Version 7 (sequence alignment tool). Additional data collection is described in the Methods section.

For manuscripts utilizing custom algorithms or software that are central to the research but not yet described in published literature, software must be made available to editors/reviewers. We strongly encourage code deposition in a community repository (e.g. GitHub). See the Nature Research guidelines for submitting code & software for further information.

Data

Policy information about availability of data

All manuscripts must include a data availability statement. This statement should provide the following information, where applicable:

- Accession codes, unique identifiers, or web links for publicly available datasets
- A list of figures that have associated raw data
- A description of any restrictions on data availability

All available PB2 sequences of influenza A were retrieved from fludb.org (https://www.fludb.org/brc/influenza_sequence_search_protein_display.spg?method=ShowCleanSearch&decorator=influenza). The datasets generated during and/or analyzed during the current study are available from the corresponding author on reasonable request.

Field-specific reporting

Please select the one below that is the best fit for your research. If you are not sure, read the appropriate sections before making your selection.

☒ Life sciences ☐ Behavioural & social sciences ☐ Ecological, evolutionary & environmental sciences

For a reference copy of the document with all sections, see nature.com/documents/nr-reporting-summary-flat.pdf

Life sciences study design

All studies must disclose on these points even when the disclosure is negative.

Sample size	A minimum sample sizes of 3 independent experiments for all experiments was used. Often, more independent experiments were performed. This is noted in each individual figure legend. For most experiments, no statistical size calculation was performed as it was impossible to estimate the effect in advance. In most of our described experiments, the effect was obvious, the sample conditions differed clearly from control conditions and 3 or more independent repeats were sufficient to show this significant effect.
Data exclusions	No data was excluded.
Replication	At least three independent repeats were performed in every experiment. All attempts at replication were successful.
Randomization	In all in vivo experiments, animals of the same age (BALC/c) and background were randomly assigned to each group (controls and peptide-treated). For in vitro experiments, randomization was not applicable.
Blinding	No blinding was used in these experiments. Blinding was not relevant for our in vivo study since differences between groups were determined by quantifiable parameters such as plaque-size reduction assays. No bias was possible.

Reporting for specific materials, systems and methods

We require information from authors about some types of materials, experimental systems and methods used in many studies. Here, indicate whether each material, system or method listed is relevant to your study. If you are not sure if a list item applies to your research, read the appropriate section before selecting a response.

Materials & experimental systems

n/a	Involved in the study	
☐	☒	Antibodies
☐	☒	Eukaryotic cell lines
☒	☐	Palaeontology
☐	☒	Animals and other organisms
☐	☒	Human research participants
☒	☐	Clinical data

Methods

n/a	Involved in the study	
☒	☐	ChIP-seq
☒	☐	Flow cytometry
☒	☐	MRI-based neuroimaging

Antibodies

Antibodies used	Goat anti-RNP Bei Resources Cat# NR-3133, Anti-goat-HRP Santa Cruz Biotechnology Cat# SC-2020, Anti-mouse-HRP ThermoFisher Cat# 62-6520, Anti-FLAG antibody Cell Signaling Cat# D6W5, Anti-vimentin antibody Santa Cruz Biotechnology Cat# SC-V9, Anti-GAPDH antibody Santa Cruz Biotechnology Cat# SC-6C5, Anti-rabbit antibody-AlexaFluor594 ThermoFisher Cat# R37117, Anti-Flavivirus Group Antigen Antibody, clone D1-4G2-4-15 Millipore Cat# MAB10216
Validation	All antibodies were obtained from commercial sources and have been heavily referenced. In addition, the antibodies were validated by immunoblotting by molecular weight.<br><br>For Goat anti-RNP: The supplier website provides a list of references that show antibody validation (https://www.beiresources.org/Catalog/BEIPolyclonalAntiserum/NR-3133.aspx):<br>- Schild, G. C. and H. G. Pereira. "Characterization of the Ribonucleoprotein and Neuraminidase of Influenza A Viruses by Immunodiffusion." J. Gen. Virol. 4 (1969): 355–363. PubMed: 4977660.<br>- NIAID. "NIAID Resources for Influenza Research". (1998): www.kamtekinc.com/pdfdoc/niaidfc.pdf.<br><br>For Anti-goat-HRP: the supplier website (https://datasheets.scbt.com/sc-2354.pdf) lists references that show antibody validation:<br>- Cernuda-Morollon, E., et al. 2002. PPAR agonists amplify iNOS expression while inhibiting NFkB: implications for mesangial cell activation by cytokines. J. Am. Soc. Nephrol. 13: 2223-2231.<br>- Ciana, A., et al. 2011. On the association of lipid rafts to the spectrin skeleton in human erythrocytes. Biochim. Biophys. Acta 1808: 183-190.<br><br>Anti-mouse-HRP: the supplier website provides specific validation examples: https://www.thermofisher.com/order/genome-

database/generatePdf?productName=Mouse%20IgG%20(H+L)&assayType=PRANT&detailed=true&productId=62-6520

Anti-FLAG antibody: the supplier website provided a list of references showing antibody validation (https://www.cellsignal.com/products/primary-antibodies/dykddddk-tag-d6w5b-rabbit-mab-binds-to-same-epitope-as-sigma-s-anti-flag-m2-antibody/14793):
- Qin G, Wang X, Ye S, et al. NPM1 upregulates the transcription of PD-L1 and suppresses T cell activity in triple-negative breast cancer. Nat Commun. 2020;11(1):1669. Published 2020 Apr 3. doi:10.1038/s41467-020-15364-z

Anti-vimentin antibody: the supplier website provided a list of references showing antibody validation (https://www.scbt.com/p/vimentin-antibody-v9):
- Cheong, ML. et al. 2019. PLoS ONE. 14: e0210765. (PMID: # 30695033)
- Jung, HY. et al. 2019. Nat. Cell Biol. (PMID: # 30804505)

Anti-GAPDH antibody: the supplier website provided a list of references showing antibody validation (https://www.scbt.com/p/gapdh-antibody-6c5):
- Marrocco, V. et al. 2019. J. Mol. Cell. Cardiol. 128: 212-226. (PMID: # 30742812)
- Jalal, S. et al. 2019. J. Cell. Sci. 132. (PMID: # 30787030)

Anti-Flavivirus Group Antigen Antibody: the supplier website provided a list of references showing antibody validation (https://www.sigmaaldrich.com/catalog/product/mm/mab10216?lang=en&region=BE):
- Dengue virus-specific and flavivirus group determinants identified with monoclonal antibodies by indirect immunofluorescence Henchal, E A, et al Am J Trop Med Hyg, 31:830-6 (1982)
- Bispidine-amino acid conjugates act as a novel scaffold for the design of antivirals that block Japanese encephalitis virus replication. Haridas, V, et al. PLoS Negl Trop Dis, 7: e2005 (2013)

Eukaryotic cell lines

Policy information about cell lines

Cell line source(s)	MDCK ATCC Cat# CCL-34, HEK 293 ATCC Cat# CRL-1573, Mouse fibroblast cell line L929 Sigma-Aldrich Cat# 85103115, CAD5 cells gift from Corinne Lasmezas (originally: CAD cell line from mouse Sigma-Aldrich Cat# 08100805).
Authentication	Cell lines were not authenticated.
Mycoplasma contamination	A Mycoplasma detection kit (Cat# M7006) was used to confirm the absence of Mycoplasma infections.
Commonly misidentified lines (See ICLAC register)	We have not used any commonly misidentified cell lines.

Animals and other organisms

Policy information about studies involving animals; ARRIVE guidelines recommended for reporting animal research

Laboratory animals	Female BALB/c wild type mice Charles River, 6-8 weeks of age at the start of the experiment. The suggested housing rules in appendix A of ETS123 june 2016 were respected. Each subgroup of mice (6 mice) were housed in an IVC cage (type GM500) with a surface area of 500 cm^2. The animals have an 14-10h day night cycle and have free access to standard maintenance food and water. Animals were housed at a constant temperature (22±1 °C) and humidity (55±10%), which was checked and recorder daily.
Wild animals	No wild animals were used in the present study.
Field-collected samples	No field collected samples were used in the present study
Ethics oversight	All protocols were approved by the Institutional ethics committee of Ghent University (Eth. Com. No. 2018-010). More information in Methods section.

Note that full information on the approval of the study protocol must also be provided in the manuscript.

Human research participants

Policy information about studies involving human research participants

Population characteristics	Blood was obtained from human volunteers by the Biobank of the Belgian Red Cross primarily for medical use. No relevant population characteristics are available.
Recruitment	All donors were volunteers for the Belgian Red Cross and all donors gave their informed consent for secondary use for scientific research.
Ethics oversight	The use of the blood in the current research was approved by the ethical board of the University Hospitals Leuven (project S60497). The samples were provided to us as pooled erythrocyte concentrate from mutliple anonymous donors.

Note that full information on the approval of the study protocol must also be provided in the manuscript.
